# Exposure measurement error in PM_2.5_ health effects studies: A pooled analysis of eight personal exposure validation studies

**DOI:** 10.1186/1476-069X-13-2

**Published:** 2014-01-13

**Authors:** Marianthi-Anna Kioumourtzoglou, Donna Spiegelman, Adam A Szpiro, Lianne Sheppard, Joel D Kaufman, Jeff D Yanosky, Ronald Williams, Francine Laden, Biling Hong, Helen Suh

**Affiliations:** 1 Department of Environmental Health, Harvard School of Public Health, Boston, Massachusetts, USA; 2 Department of Epidemiology, Harvard School of Public Health, Boston, Massachusetts, USA; 3 Department of Biostatistics, Harvard School of Public Health, Boston, Massachusetts, USA; 4 Department of Biostatistics, University of Washington, Seattle, Washington, USA; 5 Department of Environmental and Occupational Health Sciences, University of Washington, Seattle, Washington, USA; 6 Department of Public Health Sciences, Pennsylvania State University College of Medicine, Hershey, Pennsylvania; 7 U.S. Environmental Protection Agency, Research Triangle Park, North Carolina, USA; 8 Department of Health Sciences, Northeastern University, Boston, Massachusetts, USA

**Keywords:** Exposure measurement error, Fine particles, Fine particles of ambient origin, Monitoring data, Spatio-temporal models

## Abstract

**Background:**

Exposure measurement error is a concern in long-term PM_2.5_ health studies using ambient concentrations as exposures. We assessed error magnitude by estimating calibration coefficients as the association between personal PM_2.5_ exposures from validation studies and typically available surrogate exposures.

**Methods:**

Daily personal and ambient PM_2.5_, and when available sulfate, measurements were compiled from nine cities, over 2 to 12 days. True exposure was defined as personal exposure to PM_2.5_ of ambient origin. Since PM_2.5_ of ambient origin could only be determined for five cities, personal exposure to total PM_2.5_ was also considered. Surrogate exposures were estimated as ambient PM_2.5_ at the nearest monitor or predicted outside subjects’ homes. We estimated calibration coefficients by regressing true on surrogate exposures in random effects models.

**Results:**

When monthly-averaged personal PM_2.5_ of ambient origin was used as the true exposure, calibration coefficients equaled 0.31 (95% CI:0.14, 0.47) for nearest monitor and 0.54 (95% CI:0.42, 0.65) for outdoor home predictions. Between-city heterogeneity was not found for outdoor home PM_2.5_ for either true exposure. Heterogeneity was significant for nearest monitor PM_2.5_, for both true exposures, but not after adjusting for city-average motor vehicle number for total personal PM_2.5_.

**Conclusions:**

Calibration coefficients were <1, consistent with previously reported chronic health risks using nearest monitor exposures being under-estimated when ambient concentrations are the exposure of interest. Calibration coefficients were closer to 1 for outdoor home predictions, likely reflecting less spatial error. Further research is needed to determine how our findings can be incorporated in future health studies.

## Background

Exposure measurement error is a limitation of epidemiologic studies of fine particles (PM_2.5_) [1-3], which generally assess exposures using ambient concentrations measured at centrally located monitors. The impact of error on observed health risks can be substantial, potentially distorting associations and interactions between covariates and outcomes, reducing the power to detect effects, and leading to invalid inference [3-5].

In time series studies, use of measurements from ambient monitors, even in absence of any instrumental error, has been shown to introduce both a Berkson error component, a result of using aggregated instead of individual exposure data, and a classical error component, a result of the difference between the aggregated exposure data and the true ambient PM_2.5_ concentrations [5]. Berkson error would not bias the health effect estimates, but would lead to an increased variance, while classical error, conversely, can lead to bias [5,6]. It has been shown that in presence of multiple monitoring sites in a city, using across-monitor averages, or population-weighted averages, would lead to less bias in time series studies [6,7]. Furthermore, less bias is expected when the pollutant of interest is spatially homogeneous, such as PM_2.5_[6,8].

To minimize error, PM_2.5_ exposures would ideally be measured using personal monitors, with analytical methods used to apportion these measurements into PM_2.5_ constituents and sources. Such methods, however, are both expensive and intrusive and thus not feasible for studies conducted over long time periods with many subjects. Recently, for application to cohort studies, researchers have used statistical models to predict exposures outside participant residences [9-14], thus accounting for spatial variation in ambient concentrations. While an improvement, such models still do not account for all sources of exposure variability, such as activity patterns, which can lead to biased results [15]. It can be argued, nevertheless, that such “biases” are the result of different target parameters of interest for the health effects of ambient concentration vs. personal or ambient source exposure [16].

Further, spatial smoothing models can contribute a Berkson-like error component that results from smoothing the exposure surface and a classical-like component from variability in estimating model parameters [17-19]. The classical-like error can induce bias both towards and away from the null [18] with increased variability [18,20,21]. Even in absence of other error sources, nevertheless, health effects estimated using outdoor concentrations will be attenuated proportionally to the PM_2.5_ infiltration factor, the factor describing how much of the personal PM_2.5_ was generated outdoors, penetrated the building envelope and remained airborne [5]; if the exposure of interest is outdoor pollutant concentration rather than infiltrated personal exposure, however, it has been argued that this attenuation should not be regarded as a manifestation of measurement error [19].

Several previous acute effects studies have adjusted for exposure measurement error, showing that use of surrogate exposures tends to bias the health effect estimates towards the null [22-24]. For long-term PM_2.5_ effects, a limitation in understanding the impact of measurement error on estimated health risks is the paucity of long-term personal exposure data [25-28]. We compiled exposure data from nine studies to estimate calibration coefficients for PM_2.5_ of ambient origin and total personal PM_2.5_ for cases when ambient concentrations or spatial models are used to assess exposures. In light of the complexity of measurement error in air pollution, the time scale of our validation data, and the uncertainty in our estimated calibration coefficients, our aim was to estimate and characterize calibration coefficients for PM_2.5_, but not to recommend their use to adjust health effect estimates in epidemiology studies directly. Our group is currently developing statistical methods to account for these limitations.

## Methods

### Personal exposure datasets

We included data from studies of personal PM_2.5_ exposures based on the following criteria: i) the study had to be conducted in the United States, ii) during or after 1999, to ensure availability of PM_2.5_ concentration measurements at a EPA monitor located nearby, and iii) we had to be able to obtain the raw data, vs. the published summary statistics, from the investigators who originally conducted the study.

Measurements of personal and ambient PM_2.5_ and, when available, sulfate (${\text{SO}}_{4}^{2-}$), were compiled from nine cities located throughout the United States (Table 1) [29-41]. A brief description of the validation studies is presented in the Additional file 1.

**Table 1:** Validation studies used in our analyses

**Cities**	**Years**	**# Subjects**	**Sample session**	**Age**	**References**
			**duration**	**Mean (S.D.)**	
Atlanta, GA	1999–2000	31	7 days	65.0 (13.5)	[36]
Baltimore, MD	1998–1999	35	12 days	70.8 (7.7)	[30,33,37,38]
Boston, MA	1999–2000	56	7 or 12 days	62.3 (14.1)	
Los Angeles, CA	2000–2002	37	7 days	56.3 (13.9)	[34,35]
RTP, NC	2000–2001	37	7 days	64.5 (7.8)	[40,41]
RIOPA	1999-2001		48 hr		[32,39]
Los Angeles, CA		73		46.1 (18.6)	
Elizabeth, NJ		57		48.2 (17.8)	
Houston, TX		62		48.5 (16.6)	
Seattle, WA	2000–2001	89	10 days	76.7 (6.5)	[31]
Steubenville, OH	2000	28	48 hr/we for 12 we	71.0 (10.0)	[29]

In each study, daily personal PM_2.5_ exposure data were collected following panel study sampling designs. The number of subjects per study ranged between 15–201, with sampling session durations ranging from 2 to 12 days (median: 7 days). For each subject, we estimated monthly average personal exposures and used these in our analyses.

All subjects were non-smokers and were monitored in multiple seasons. Study subjects included the elderly, patients with myocardial infarction, children, and adults. All subjects younger than 18 years were excluded from the analysis, since long-term air pollution health studies are often focused on adult mortality.

The current analysis was approved by the Human Subjects Committee of the Harvard School of Public Health. All participants provided informed consent according to the protocols of the original studies.

### Surrogate exposures

For each subject, we calculated two monthly PM_2.5_ surrogate exposures. First, we determined monthly ambient PM_2.5_ concentrations from the nearest US Environmental Protection Agency (EPA) Air Quality System monitor (nearest monitor), restricting the maximum allowed monitor-residence distance to 30 mi [42]. Monthly concentrations were estimated using all available data within the month, i.e. not only the days used for the monthly averages of the personal exposures.

Second, we estimated monthly outdoor PM_2.5_ concentrations outside each subject’s residence, at the latitude and longitude of each subject’s residence (at the zip code level for RIOPA subjects), using a nationwide expansion of a geographic information system (GIS)-based spatio-temporal model [14,43]. This model predicts monthly PM_2.5_ concentrations using a generalized additive model that fits monitoring data from governmental and research networks together with GIS-based covariates, including population density, distance to nearest roads, elevation, urban land use, PM_2.5_ point-source emissions and weather variables.

### Estimation of personal exposures of ambient origin

We assume the true exposure metric is personal exposures to PM_2.5_ of ambient origin, which reflect PM_2.5_ from sources relevant to epidemiological studies of ambient air pollution [26,44,45]. This quantity cannot be measured directly.

To estimate personal PM_2.5_ of ambient origin, we used ambient ${\text{SO}}_{4}^{2-}$ measurements, which were available for four cities (Atlanta, Baltimore, Boston and Steubenville). The majority of ${\text{SO}}_{4}^{2-}$ is formed in the atmosphere through secondary reactions via either gas-phase or gas/particle phase oxidation [46] and is generally associated with coal combustion and coal-fired power plant emissions [47,48]. Because of negligible indoor sources and its similar spatial homogeneity as PM_2.5_, ${\text{SO}}_{4}^{2-}$ can serve as a tracer for PM_2.5_ of ambient origin in locations where ${\text{SO}}_{4}^{2-}$ comprises a large part of the PM_2.5_ mass [49,50], with personal to ambient ${\text{SO}}_{4}^{2-}$ ratio approximating the fraction of ambient PM_2.5_ that infiltrates indoors and remains airborne:

$${\text{PM}}_{\text{pers. of ambient origin}}=\frac{{\text{SO}}_{4_{\;\;\text{pers.}}}^{2-}}{{\text{SO}}_{4_{\;\;\text{ambient}}}^{2-}}{\text{PM}}_{\text{ambient}}$$

In Seattle, for which personal ${\text{SO}}_{4}^{2-}$ data were not available, personal PM_2.5_ of ambient origin was estimated as the weighted average of the indoor PM_2.5_ of ambient origin (estimated using the corresponding calculated home infiltration efficiency) and ambient PM_2.5_, with the proportion of time each subject spent indoors and outdoors as weights [51].

Since personal exposures to PM_2.5_ of ambient origin could only be estimated in five cities, we also assessed error using total personal PM_2.5_ exposure. For this measure, calibration coefficients will be less accurate, since total personal PM_2.5_ exposures also include indoor- and personally-generated PM_2.5_, which are independent from ambient PM_2.5_[45].

### Calibration coefficients

The calibration coefficients were estimated as the fixed regression coefficients (*γ*_1_) from linear mixed effects models, of monthly averaged “true” on surrogate exposures, accounting for within-city correlated observations and repeated measures within subject:

(1) $$\;X_{\text{ijk}}=(\gamma_{0}+g_{1i}+g_{2\text{ij}})+(\gamma_{1}+g_{3i})Z_{\text{ijk}}+\gamma_{2}{\text{Season}}_{\text{ijk}}+\varepsilon_{\text{ijk}},$$

where *X*_*ijk*_ are the “true” (either personal PM_2.5_ of ambient origin or total personal PM_2.5_) and *Z*_*ijk*_ the surrogate exposures (either nearest ambient PM_2.5_ monitor or spatio-temporal model predictions) for *j*=1, ⋯,*J*_*i*_ subjects within city *i*= 1,⋯,*I*, and *I*=5 or 9, with *k*=1, ⋯,*K*_*ij*_ repeated measures, $g_{1i}∼N(0,\sigma_{\text{city}}^{2}),\;g_{2\text{ij}}∼N(0,\sigma_{\text{subject}}^{2}),\;g_{3i}∼N(0,\sigma_{\text{CF-city}}^{2})$ and $\varepsilon_{\text{ijk}}∼N(0,\sigma_{W}^{2})$.

We explored the sensitivity of our results to assumptions about the covariance structure for repeated measures within subjects. Results are reported assuming compound symmetry covariance, with results similar for autoregressive covariance structure or when allowing heteroscedasticity. We also allowed for random seasonal effects by city, but our results were materially unchanged (results not shown).

Calibration coefficients equal to 1 suggest no bias, while coefficients <1 suggest an attenuated effect estimate. The p-values (as p-value_1_) presented with the estimated calibration coefficients correspond to the hypothesis that *γ*_1_=1 and were obtained using $\frac{{\left({\hat{\gamma }}_{1}-1\right)}^{2}}{\hat{\text{var}}({\hat{\gamma }}_{1})}∼\chi_{1}^{2}$.

Potential effect modification by season, with October–March as winter and April–September as summer, was assessed, as the association between personal exposures and ambient concentrations differs by season [29,33,37,38]. Stratified calibration coefficients are presented when the estimated interaction term for season was significant. Statistical significance was assessed at the 0.05 level.

### Between–city heterogeneity

To assess potential between-city heterogeneity in the calibration coefficients, we tested the hypothesis *H*_0_: $\sigma_{\text{CF-city}}^{2}$ = 0, comparing Model 1 to Model 2, where Model 2 is the same as Model 1 without the random slope for cities (*g*_3*i*_):

(2) $$X_{\text{ijk}}=(\gamma_{0}+g_{1i}+g_{2\text{ij}})+\gamma_{1}Z_{\text{ijk}}+\gamma_{2}{\text{Season}}_{\text{ijk}}+\varepsilon_{\text{ijk}}$$

We used a likelihood ratio test (LRT) for this comparison, with LRT ∼ 50:50 mixture of $\chi_{0}^{2}$ and $\chi_{1}^{2}$ and p-value = 0.5 if ${\hat{\sigma }}_{\text{CF-city}}^{2}=0$ and p-value = 0.5$\times (1-\chi_{1}^{2}\times (\text{LRT}))$ otherwise [52].

We used step-wise selection to identify city-specific variables explaining any observed between-city heterogeneity in the calibration coefficients. In presence of significant heterogeneity, we added to Model 1 candidate city-specific variables together with interaction terms between the candidate variable and the surrogate exposure (Model 3). The candidate variables were kept in the model if the interaction term was significant.

(3) $$\begin{array}{ll} X_{\text{ijk}} & =(\gamma_{0}+g_{1i}+g_{2\text{ij}})+(\gamma_{1}+g_{3i})Z_{\text{ijk}}+\gamma_{2}{\text{Season}}_{\text{ijk}}\\ & \;+\gamma_{3}{\text{CityVariables}}_{i}+\gamma_{4}Z_{\text{ijk}}{\text{CityVariables}}_{i}+\varepsilon_{\text{ijk}} \end{array}$$

Candidate city-specific variables were identified from previous studies showing their importance to the personal-ambient relationship, including air conditioning use, unemployment, race, public transport [53-55] and traffic [54] (Additional file 1: Table S4). City-specific variables were obtained from the U.S. Census Bureau (Census 2000, http://www.census.gov), the American Housing Survey (http://www.census.gov/programs-surveys/ahs/), the National Climatic Data Center (http://www.ncdc.noaa.gov) and the Bureau of Labor Statistics (http://www.bls.gov).

Leave-one-out cross-validation techniques were employed to validate the variable selection process [56, Chapter 7.10]. By omitting one city at a time (−*i*), we re-fit Model 3, using data from the remaining *I*−1 cities, allowing for a different set of variables to be selected each time. We then predicted the city-specific calibration coefficient for the omitted city using the estimated model parameters together with the selected variable(s) of the omitted city, i.e. ${\hat{\gamma }}_{1i-}={\hat{\gamma }}_{1(-i)}+{\hat{\gamma }}_{4(-i)}{\text{CityVariables}}_{i}$. We also estimated city-specific calibration coefficients (${\hat{\gamma }}_{1i}$) employing city-specific mixed effects models (Model 4). Finally, we compared the predicted to the observed city-specific calibration coefficients obtained from the city-specific models.

(4) $$X_{\text{ijk}}=(\gamma_{0i}+g_{2\text{ij}})+\gamma_{1i}Z_{\text{ijk}}+\gamma_{2i}{\text{Season}}_{\text{ijk}}+\varepsilon_{\text{ijk}}$$

We assessed the cross-validated results by the correlation between the predicted (${\hat{\gamma }}_{1i-}$) and observed (${\hat{\gamma }}_{1i}$) calibration factors, the relative bias $\frac{{\hat{\gamma }}_{1i-}-{\hat{\gamma }}_{1i}}{{\hat{\gamma }}_{1i}}$ and the absolute bias $\left|\frac{{\hat{\gamma }}_{1i-}-{\hat{\gamma }}_{1i}}{{\hat{\gamma }}_{1i}}\right|$, both averaged over all cities.

### Sensitivity analyses

To assess the robustness of our results, we assessed potential effect modification by subpopulation: seniors (subjects older than 65 years old) and subjects with COPD, myocardial infarction (MI), and coronary heart disease (CHD).

Sensitivity analyses were also performed to assess the effect of imperfectly matched monthly ambient and personal exposures. We calculated calibration coefficients for monthly ambient levels estimated using only those days for which personal exposure measures were available. Since the EPA does not collect data daily at all locations, we allowed subjects to be matched to the nearest monitor with available data for that day. This sensitivity analysis could only be performed for the nearest ambient monitor concentrations, as the outdoor home model predictions were calculated at the monthly level only.

In addition, we calculated calibration coefficients for total personal PM_2.5_ exposures using the identical data as used to calculate calibration coefficients for personal PM_2.5_ of ambient origin.

All statistical analyses were conducted using SAS software (Version 9.3, SAS Institute Inc, Cary, NC).

## Results

Summary statistics and ambient-personal correlations are presented in Table 2 and Additional file 1: Table S2, respectively. By-city summary statistics are presented in Additional file 1: Table S1, and the relationship between exposure to PM_2.5_ of ambient origin and ambient PM_2.5_ concentrations is presented in Additional file 1: Figure S1. On average, total personal PM_2.5_ was higher than both concentrations at the nearest ambient monitor and outdoor home predictions. Concentrations at the ambient monitors were strongly correlated with outdoor home model predictions (Spearman *r*_*s*_=0.86). PM_2.5_ of ambient origin contributed 62%, on average, to the total personal PM_2.5_.

**Table 2:** Basic characteristics of exposure variables

	**# person-months**	**Mean *± *** **SD**
	**(# subjects)**	**( *μ *** **g/m**^***3***^**)**
*All Year*		
Total Personal PM_2.5_	919 (490)	24.54 ± 18.97
Personal PM_2.5_ of ambient origin	261 (141)	9.71 ± 4.32
Personal/Ambient ${\text{SO}}_{4}^{2-}$ ratio ^a^	241 (131)	0.64 ± 0.25
Model Predicted PM_2.5_	1029 (502)	15.47 ± 4.77
Monitor PM_2.5_	1029 (502)	15.86 ± 5.58
*Summer*		
Total Personal PM_2.5_	429 (312)	23.92 ± 16.92
Personal PM_2.5_ of ambient origin	130 (90)	10.95 ± 4.12
Personal/Ambient ${\text{SO}}_{4}^{2-}$ ratio ^a^	125 (86)	0.70 ± 0.23
Model Predicted PM_2.5_	493 (327)	15.67 ± 4.84
Monitor PM_2.5_	493 (327)	15.69 ± 4.79
*Winter*		
Total Personal PM_2.5_	490 (353)	23.94 ± 19.67
Personal PM_2.5_ of ambient origin	131 (97)	8.73 ± 4.27
Personal/Ambient ${\text{SO}}_{4}^{2-}$ ratio ^a^	116 (87)	0.59 ± 0.24
Model Predicted PM_2.5_	536 (367)	15.36 ± 4.71
Monitor PM_2.5_	536 (367)	16.10 ± 6.21

^a^Not estimated for Seattle, WA.

### Calibration coefficients

The results from the linear mixed effects model (Model 1) for both personal PM_2.5_ of ambient origin and total personal PM_2.5_ are presented in Table 3.

**Table 3:** **Season-adjusted calibration factors for personal PM**_**2.5**_** of ambient origin and total personal PM**_**2.5**_

	**Monitor PM**_**2.5**_	**Model predicted PM**_**2.5**_
*PersonalPM*_2.5_*of ambient*	5 cities (141 subjects)	
*origin*		
Estimate (95% CI)^a^	0.31 (0.14, 0.47)**	0.54 (0.42, 0.65)**
p-value for between-city	0.0034	0.1114
heterogeneity		
${\text{R}}_{I}^{b}$	10.75%	0.96%
Estimate (95% CI)^c^	N/A	0.56 (0.44, 0.68)**
*Total PersonalPM*_2.5_	9 cities (490 subjects)	
Estimate (95% CI)^a^	0.56 (0.24, 0.88)**	0.81 (0.49, 1.12)
p-value for between-city	0.0084	0.1712
heterogeneity		
${\text{R}}_{I}^{b}$	2.54%	1.28%
Estimate (95% CI)^c^	N/A	0.79 (0.54, 1.04)**

*p-value_1_ < 0.05, **p-value_1_ < 0.01 for significant difference from 1.

^a^Results from Model 1 (including random slopes for cities, *g*_3*i*_).

^b^R_*I*_: the proportion of variance explained by the between-cities heterogeneity.

^c^Results from Model 2, when no significant between-city heterogeneity was detected (without the random slopes for cities, *g*_3*i*_).

When the nearest ambient monitor was used as the surrogate exposure, the calibration coefficient for personal PM_2.5_ of ambient origin was estimated as 0.31 ((95% CI:0.14, 0.47), p-value _1_<0.0001), when adjusted for seasonal effects. We found no significant seasonal effect modification (p-value = 0.71). The season-adjusted calibration coefficient was higher for outdoor home model predictions, as compared to nearest monitor PM_2.5_, equaling 0.54 (95% CI:0.42, 0.65, p-value _1_<0.0001). We found significant effect modification by season for outdoor home model predictions (p-value = 0.006), with season-stratified calibration coefficients higher during winter (0.60 (95% CI:0.36, 0.64)) than summer (0.50 (95% CI:0.42, 0.78)).

Total personal PM_2.5_ exposure calibration coefficients were higher than those for personal PM_2.5_ of ambient origin (Table 3). For total personal PM_2.5_ exposures, the season-adjusted calibration coefficient for the nearest ambient monitor was 0.56 (95% CI:0.24, 0.88, p-value_1_ = 0.007). Effect modification by season was significant (p-value = 0.041), with higher season-stratified calibration coefficients during summer (0.78 (95% CI:0.36, 1.19)) than winter (0.48 (95% CI:0.12, 0.83)). The corresponding calibration coefficient, using outdoor home model predicted PM_2.5_ as the surrogate exposure, was higher, 0.81 (0.49, 1.12, p-value_1_ = 0.234). There was no significant seasonal effect modification.

### Between–city heterogeneity

For both personal PM_2.5_ of ambient origin and total personal PM_2.5_ calibration coefficients, we found no statistically significant evidence of heterogeneity across cities for outdoor home model predictions (p-values = 0.11 and 0.17, respectively) and therefore results from Model 2, instead of Model 1, can be used. For personal PM_2.5_ of ambient origin and total personal PM_2.5_, calibration coefficients equaled 0.56 (0.44, 0.68) and 0.79 (0.54, 1.04), respectively. Since no between-city heterogeneity was detected, no further adjustment to these calibration coefficients was done.

**Figure 1:**
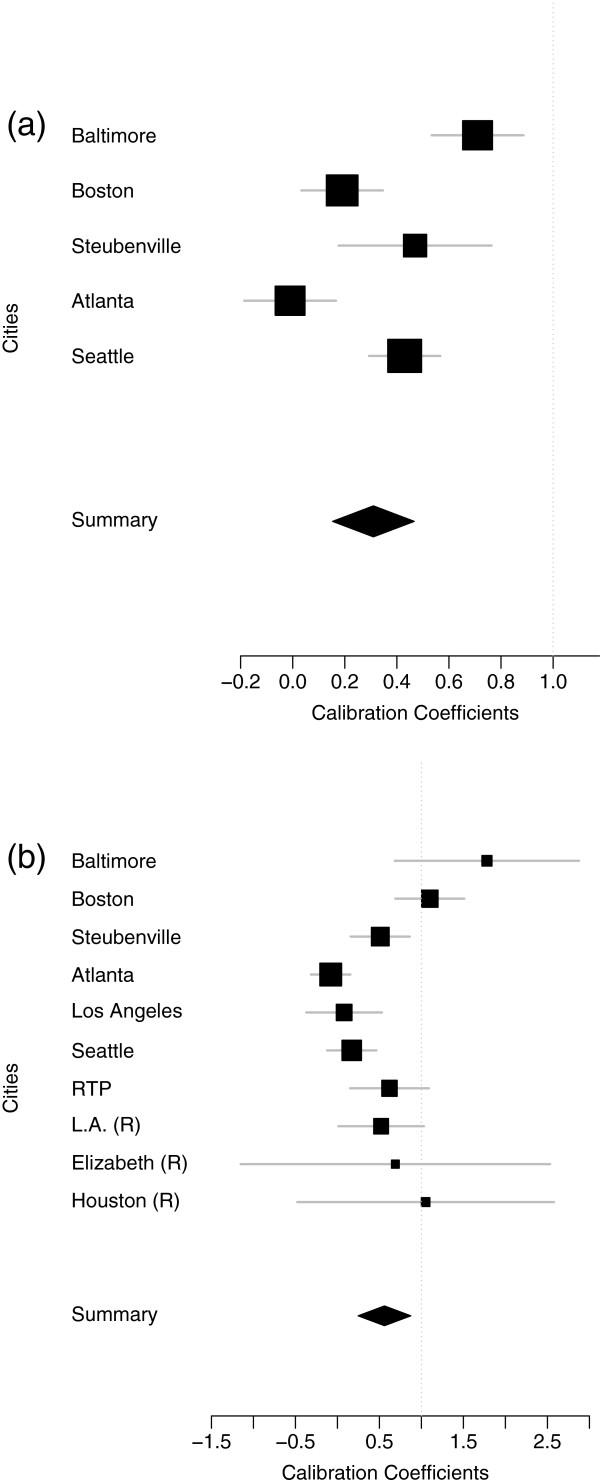
**Forest plots of the by-city calibration coefficients for (a) personal PM**_**2.5**_** of ambient origin and (b) total personal PM**_**2.5**_** and nearest monitor concentrations.** The size of the point used for the effect estimate is proportional to the precision of that calibration coefficient.

Significant between-city heterogeneity (p-value = 0.003) was detected in the calibration coefficients for personal PM_2.5_ of ambient origin, when the nearest monitor was used as the surrogate exposure, with estimated city-specific calibration coefficients ranging between 0.0-0.71 (Figure 1(a)). The observed between-city heterogeneity was explained by two variables: the city’s average number of residents in a housing unit and the city’s 30-year average of annual heating degree days, an indicator of the typical number of heating days in a year (p-value = 0.50 for the test for residual heterogeneity). Cross-validation showed, however, that these variables were not robust predictors of the between-city variation in the calibration coefficient (Additional file 1: Figure S2).

Significant between-city heterogeneity in the calibration coefficient was also detected for total personal PM_2.5_ when the nearest monitor was used as the surrogate measure (p-value = 0.008). Using Model 4, estimated city-specific coefficients ranged between 0.0-1.78 (Figure 1(b)). Step-wise selection found that some of the observed between-city heterogeneity was explained by the average number of vehicles per housing unit in each city (p-value = 0.221 for the test for residual heterogeneity). The effect of the city average vehicles per housing unit on the relationship between total personal PM_2.5_ and nearest ambient monitor PM_2.5_ concentrations was -2.53 (SE: 0.82), implying that as the average number of vehicles per housing unit increases, the calibration coefficient decreases for cities with larger numbers of vehicles per housing unit. For instance, if the average number of vehicles per housing unit in a city increased by 0.1, then the calibration coefficient for that city would decrease by 0.25. The selection of this variable was confirmed in the cross-validation, as it was consistently selected when cities were omitted one by one (Additional file 1: Figure S2). The correlation between the predicted calibration coefficients from each city and the observed by-city coefficients was 0.62 (p-value = 0.05), the mean percent relative bias was estimated -0.76% and the mean percent absolute bias 149%.

### Sensitivity analyses

Results from our sensitivity analyses are presented in the Additional file 1. Briefly, we observed no significant effect modification by subpopulation. We found significant effect modification by age, with subjects younger than 65 years of age having lower calibration coefficients than their older counterparts (Additional file 1: Table S3).

Further, we found that estimated calibration coefficients were similar irrespective of the method used to calculate monthly ambient concentrations at the nearest monitor. When all days within the month were used in the calculation, the calibration coefficient for personal PM_2.5_ of ambient origin was 0.31 (95% CI:0.14, 0.47), vs. 0.35 (95% CI:0.26, 0.43) when monthly ambient concentrations were calculated using only those days with personal monitoring.

## Discussion

We estimated calibration coefficients for studies of the association of long-term PM_2.5_ health effects with ambient air pollution exposures, considering both estimated personal exposures to PM_2.5_ of ambient origin as the exposure metric and personal exposures to total PM_2.5_ as a second, albeit imperfect, exposure metric. Our goal was to assess and quantify error resulting from use of surrogate exposures and characterize the impact of different surrogate exposures on error. As discussed in the introduction, nevertheless, the estimated error could be from a variety of sources, and it has been argued that not all of these are properly characterized as measurement error [19].

Using estimated monthly personal PM_2.5_ of ambient origin from five cities as the true exposure measure, we estimated a calibration coefficient of 0.54 (95% CI:0.42, 0.65) when outdoor home model predictions were used as the surrogate exposure, with no city-specific heterogeneity. This calibration coefficient suggests that when the parameter of interest is the health effect of ambient source pollution, the observed effect could be half the true estimate when outdoor home model predictions are used as the exposure metric in a linear health model, in absence of other potential bias sources. The lack of observed between-city variability likely reflects the use of the spatio-temporal model, which incorporates variables that may explain much of the between-city variability, such as population density, urban land use and distance to nearest road.

The estimated calibration coefficient for nearest ambient monitor concentrations as the exposure metric was lower (0.31 (95% CI:0.14, 0.47) compared to 0.54 (95% CI:0.42, 0.65) for outdoor model concentrations), reflecting the fact that nearest monitor concentrations do not account for as much spatial variability in ambient concentrations as the outdoor home model predictions. We also detected statistically significant between-city heterogeneity. Factors explaining between-city variability in the calibration coefficient, nevertheless, could not be reliably identified. This inability to explain the city-specific heterogeneity likely reflects the small number of cities included in our analysis.

When total PM_2.5_ was used as the true exposure measure, calibration coefficients of 0.56 (95% CI:0.24, 0.88) and 0.81 (95% CI:0.49, 1.12) were found for nearest ambient monitor PM_2.5_ and outdoor home model predictions, respectively. These results are consistent with those reported in Setton et al. (2011) [15], who reported an attenuation ranging between 0.70 to 0.84 for scenarios when mobility was not considered and only PM_2.5_ predictions at the subjects’ residences were included in the health model. As noted above, however, these calibration coefficients were calculated using total personal PM_2.5_, an imperfect measure of true exposure to ambient-generated pollutants.

As was the case with personal PM_2.5_ of ambient origin, we detected significant between-city heterogeneity in total PM_2.5_ calibration coefficients only when nearest monitor concentrations were used as the surrogate exposure. For nearest monitor PM_2.5_, between-city heterogeneity was explained with the city average number of vehicles per housing unit. Results showed that error increases with vehicles per housing unit. A possible explanation for this association is provided by the strong negative correlation between the number of average vehicles per housing unit and population density (*r*=−0.86) and the strong positive correlation with the percentage of the detached homes in a study area (*r*=0.88) as shown in Additional file 1: Figure S3. These correlations suggest that in less dense cities, residents need to travel longer distances, possibly increasing the impact of pollutant spatial variability. These results are also in agreement with Setton et al. (2011) [15], who found increasing bias with increasing distance spent away from home. Selection of number of vehicles per housing unit to explain between-city heterogeneity could also reflect varying PM_2.5_ composition, with local sources, such as traffic, likely comprising a larger portion of PM_2.5_ mass in cities with more vehicles per housing unit, than regional sources. PM_2.5_ of local sources is more spatially heterogeneous and more error is, therefore, expected when it comprises a large fraction of the total ambient PM_2.5_. The fact, however, that our estimated city-specific calibration coefficients ranged between 0.0-1.9 complicates our interpretation of the overall estimate of 0.56 (95% CI:0.24, 0.88) and of the observed association with housing and transportation characteristics, suggesting that one average calibration coefficient may not adequately describe error from use of ambient monitor measurements across the United States.

Environmental tobacco smoke (ETS) may also contribute, at least partially, to the observed between-city heterogeneity. In all studies in our analyses, subjects were selected as non-smokers, living in non-smoking homes. Although this inclusion criterion would minimize potential exposure to ETS, it is possible that participants living in cities with more ETS would also have higher personal PM_2.5_ exposures, thereby potentially contributing to between-city heterogeneity in the calibration coefficients. We, however, were not able incorporate ETS exposures in our analysis, as some studies did not report ETS exposure information.

Our findings are consistent with two studies by Avery et al. (2010) [57], who found a median correlation coefficient of 0.54 between total personal PM_2.5_ exposures and concentrations at a centrally located monitor, and strong between-city heterogeneity (p-value <0.0001). Although their reported median correlation coefficient between total personal PM_2.5_ exposures and outdoor home concentrations was similar, between-city heterogeneity in this association was lower (p-value = 0.05). The weaker evidence of heterogeneity for outdoor home PM_2.5_ concentrations is consistent with our suggestion that between-city heterogeneity in calibration coefficients is explained by variables included in outdoor home model predictions; this is one explanation for why we found heterogeneity only for nearest monitor but not outdoor home exposures.

Our study is limited by several factors. First, the data available to validate the exposure metrics of interest were limited to a small number of cities and participants, especially for personal PM_2.5_ of ambient origin. Also, only a small number of days in a month were available in some cities to estimate monthly averages. These small numbers contributed to uncertainty in our data and estimates, and potentially prohibited detection of any potential between-city heterogeneity for outdoor home predictions and the identification of factors explaining observed between-city heterogeneity in calibration coefficients when the nearest monitor PM_2.5_ concentrations were the exposure surrogate. Further, the cities included in our analyses may not be representative of all US cities, and thus our estimated calibration coefficients might not be generalizable to other cities. Moreover, the association between personal exposures and ambient concentrations might vary over years. Since our studies were conducted over a one to two year time span (Table 1), we were not able to assess the contribution of longer term personal-ambient trends to total error.

In addition, personal PM_2.5_ of ambient origin was estimated rather than measured. As a result, estimated exposures did not take into account the uncertainty related to their prediction when estimating the calibration coefficients. Moreover, given data availability, we were not able to estimate the contribution of instrumental to total error. Both personal and ambient measurements are prone to instrumental error, presence of which is likely to introduce classical error [5]. In our setting, however, personal exposures are the outcome variable in the regression and therefore random error in these exposures is not expected to introduce error in the estimated calibration coefficients. Furthermore, personal exposures are on average measured with high precision and accuracy [29,30].

To estimate personal PM_2.5_ of ambient origin we used the ${\text{SO}}_{4}^{2-}$ tracer method. In cities where ${\text{SO}}_{4}^{2-}$ comprises a large fraction of the total ambient PM_2.5_ mass, as in the northeastern US [58], the ${\text{SO}}_{4}^{2-}$ tracer method has been shown to perform well [49]. In places, however, where ambient PM_2.5_ mass is strongly influenced by local sources, such as traffic, ambient ${\text{SO}}_{4}^{2-}$ would not act as good tracer, given that the spatial and size distributions of ${\text{SO}}_{4}^{2-}$ may differ from those of PM_2.5_. Since PM_2.5_ from local sources is more spatially heterogeneous, larger spatial misalignment would be expected in these cities and, hence, more measurement error. For these cities, we would expect the calibration coefficients for personal PM_2.5_ of ambient origin, which was estimated using the ${\text{SO}}_{4}^{2-}$ ratio, to be overestimated and the error to be underestimated, a factor likely contributing to the observed between-city heterogeneity. In our study, we only had ${\text{SO}}_{4}^{2-}$ data in four cities, three of which are in the northeastern US (Baltimore, Boston and Steubenville). The fourth city was Atlanta, which has been shown, on average, to have lower ${\text{SO}}_{4}^{2-}$ concentrations [58]. Even there, however, secondary sulfate was found to comprise 38% of the total PM_2.5_ mass [59] and in our data, the ratio of ambient ${\text{SO}}_{4}^{2-}$ over PM_2.5_ in Atlanta was, on average, similar to the ratios in the three northeastern cities (Additional file 1: Table S1).

In addition, we estimated the outdoor home predictions using a specific spatio-temporal model. This model has been validated and shown to perform very well [14,43]. We would therefore expect that our findings for outdoor home predictions could be extended to similarly performing spatio-temporal models and could be qualitatively used for predicted concentrations obtained from other spatio-temporal models.

Moreover, we were not able to disentangle how specific error types would impact the health effect estimates obtained using either of the surrogate exposures. We did not assume models addressing specific error structures and our approach assesses overall error from use of surrogate exposures, combining the multiple error types that are likely present [5,18].

Furthermore, our study is not able to determine how much of the estimated calibration coefficient reflects infiltration of particles from outdoor to indoor environments, as compared to other sources of the difference between personal exposure and outdoor concentration metrics [5,19]. Infiltration, however, does not appear to explain all of the observed error found in our analysis, since the average estimated calibration coefficients for personal PM_2.5_ of ambient origin were <0.64 (the approximated penetration efficiency using the ${\text{SO}}_{4}^{2-}$ ratio), consistent with additional contributing error sources.

Additionally, personal exposures were measured for each participant for periods less than one month. We would expect this temporal mismatch to introduce both Berkson, through the errors in the true exposures that were randomly selected within a month, and classical, through the errors in the temporal misalignment of the surrogate exposures, error components. Through sensitivity analyses, comparing PM_2.5_ concentrations measured at the nearest monitor using all data within a month with that measured on days when personal data were also available, we showed the point estimates to be very similar, but the confidence intervals for the calibration coefficients estimated using the temporally mismatched data were wider. Since outdoor home model predictions were only available at the monthly level, we were unable to quantitatively assess the effect of this temporal mismatch on the estimation of the calibration coefficients. Monthly concentrations at the nearest ambient monitor, however, were very strongly correlated with outdoor home model predictions (*r*=0.86). In any event, randomly temporally mismatched data relating personal exposures to outdoor home predictions may also lead to increased uncertainty, but likely no bias, in the calibration coefficients.

Finally, our goal was to assess exposure measurement error in long-term PM_2.5_ exposures. As described earlier, personal exposure studies are infeasible for long periods and, given current data availability, we were only able to conduct our analyses using monthly averages. Many long-term PM_2.5_ studies use exposure metrics based on functions of monthly averages (e.g. 12-month moving average [11] or cumulatively-updated monthly average [60]), and we therefore believe that our findings provide useful information in the interpretation of chronic health effect estimates.

We compiled data from 9 cities across the United States for our analyses and calculated calibration coefficients that may be informative for interpreting risk estimates in nationwide studies of long-term PM_2.5_ health effects. For instance, differential measurement error could be partially responsible for the higher effects reported by Puett et al. (2009) [11], who used PM_2.5_ predictions outside the participant’s homes, as compared to the effects found by Krewski et al. (2005) [61], who used metropolitan area means of PM_2.5_ concentrations at ambient monitors.

To our knowledge, this is the first study to assess error due to two different, widely used, surrogate exposures, using personal exposure data from multiple US cities. Further, we identified variables explaining the heterogeneity in the calibration coefficients across cities, with the variances of the reported calibration coefficients potentially reflecting this heterogeneity.

At this time, we do not recommend using the calibration coefficients reported here to directly adjust health effect estimates in epidemiology studies. Given the observed between-city heterogeneity, the complex, time-varying nature of the exposures and the lack of information on individual characteristics, which would be included as confounders in health models, standard error correction methods such as ordinary regression calibration could still yield biased estimates [62,63]. Our group is currently developing methods to account for the above limitations in order to correctly adjust health effect estimates obtained using surrogate exposures. Furthermore, future research on PM_2.5_-related measurement error should characterize measurement error for regional and local PM_2.5_ by focusing on PM_2.5_ composition, which changes both over space and time, suggesting that calibration coefficients will also change over space and time [6,8,48].

## Conclusions

With our study we were able to assess the ability of two widely used surrogate exposures to reflect personal exposures: ambient concentrations measured at centrally located monitors, as well as outdoor home predictions. Our estimated calibration coefficients are consistent with previously reported chronic health risks using nearest monitor exposures being under-estimated when ambient concentrations were the exposure of interest. For outdoor home predictions, our results suggest less error.

## Abbreviations

CHD: Coronary heart disease; COPD: Chronic obstructive pulmonary disease; EPA: Environmental protection agency; MI: Myocardial infarction; PM2.5: Fine particulate matter; ${\text{SO}}_{4}^{2-}$: Sulfate.

## Competing interests

The authors declare that they have no competing interests.

## Authors’ contributions

MAK was responsible for design, conduct, analysis, interpretation of findings and writing the manuscript; DS for conception and design; AAS for interpretation of findings and manuscript review; LS and JDK for compilation of data and interpretation; JDY for providing the spatio-temporal model predictions and manuscript review; RW for compilation of data and manuscript review; FL for interpretation; BH for analysis; and HHS for conception, design, compilation of data and drafting the manuscript. All authors read and approved the final manuscript.

## Supplementary Material

Additional file 1 Supplemental material. Click here for file
