# Supplementary material for: Exposure measurement error in PM2.5 health effects studies: A pooled analysis of eight personal exposure validation studies
Source: Environ Health. 2014 Jan 13;13:2. doi: 10.1186/1476-069X-13-2 (PMC3922798; doi:10.1186/1476-069X-13-2)
Supplement: Additional file 1 — Supplemental material. [file 1476-069X-13-2-S1.pdf]

Supplemental Material

Exposure Measurement Error in $PM_{2.5}$ Health Effects Studies: A Pooled Analysis of Eight Personal Exposure Validation Studies

Marianthi-Anna Kioumourtzoglou, Donna Spiegelman, Adam A. Szpiro, Lianne Sheppard, Joel D. Kaufman, Jeff D. Yanosky, Ronald Williams, Francine Laden, Biling Hong, Helen H. Suh

Contents

S1. Personal Exposure Datasets **3**

S2. Sensitivity Analyses **4**

List of Tables

S1 By City Summary Statistics of the Exposure Variables. Concentrations are presented in μg/m^3 5

S2 Spearman correlation coefficients between exposure variables 6

S3 Stratified calibration coefficients by season and age 7

S4 Summary statistics for the variables considered to explain between-city heterogeneity, averaged across the cities included in our analyses 10

List of Figures

S1 Relationship between monthly personal $PM_{2.5}$ of ambient origin and $PM_{2.5}$ at EPA's monitors (top) and spatio-temporal model $PM_{2.5}$ predictions (bottom). 8

S2 Cross-validation results for (a) personal $PM_{2.5}$ of ambient origin and (b) total personal $PM_{2.5}$ and nearest monitor concentrations. The gray line represents perfect prediction. The observed city-specific calibration coefficients are on the x-axis, while the predicted city-specific calibration coefficients from the leave one city out procedure are on the y-axis. 9

S3 Relationship between the city-average number of cars per housing unit and the population density (left) and the percentage of detached homes in the study area (right). 11

S1. Personal Exposure Datasets

Measurements of personal and ambient $PM_{2.5}$ and, when available, sulfate (SO_4^{2-}), were compiled from nine cities located throughout the United States (Table 1) [1–13]. In each study, daily personal $PM_{2.5}$ exposure data were collected following panel study sampling designs. The number of subjects per study ranged between 15–201, with sampling session durations ranging from 2–12 days.

Briefly, all subjects were recruited at senior or community centers, through doctors referrals or advertisements. All subjects were non-smokers, living in non-smoking homes. Latitude and longitude for the addresses of the participants, zip-codes or census blocks were available. Because the goals of the validation studies were different, some studies sampled specific population groups, such as the the elderly, patients with MI, COPD or CHF, children, and adults. All subjects younger than 18 years were excluded from our analyses, since long-term air pollution health studies are often focused on adult mortality.

For all studies, personal $PM_{2.5}$ exposures were measured with Personal Environmental Monitors (PEMs), which included small inertial impactors for $PM_{2.5}$ collection on Teflon filters. All personal samplers were attached to pumps placed in carrying bags. Subjects had to keep the monitors within their breathing zones at all times, except when showering or using the restroom. During prolonged times of inactivity (such as sleeping or watching TV), subjects were allowed to remove the carrying bag from their body, but were instructed to keep the monitors as close as possible to their breathing zones. Personal SO_4^{2-} measurements were collected for four cities (Baltimore, MD, Boston, MA, Steubenville, OH and Atlanta, GA). For Baltimore and Boston the $PM_{2.5}$ filters were analyzed for SO_4^{2-} with ion chromatography, while for Steubenville and Atlanta, SO_4^{2-} were collected on fluoropore filters that were then analyzed also via ion chromatography.

S2. Sensitivity Analyses

In the main analysis for personal $PM_{2.5}$ of ambient origin we included 35 subjects with COPD and 12 subjects with MI, while in the analysis for total personal $PM_{2.5}$ we included 88 subjects with COPD, 12 subjects with MI and 21 subjects with CHD. Senior subjects accounted for the 54.4% of the total number of subjects in the 9 cities and 63.6% in the five cities with available personal $PM_{2.5}$ of ambient origin exposures. We conducted sensitivity analyses to assess whether subpopulation status modifies the estimated calibration coefficients.

We found no effect modification of the association between any true and surrogate exposures by COPD (p-value range: 0.254 – 0.939) or CHD (p-value range: 0.214 – 0.663) status. For individuals with MI, we found a significantly lower calibration coefficient for personal $PM_{2.5}$ of ambient origin in relation to the nearest ambient monitor concentrations (p-value < 0.001), but not for total personal $PM_{2.5}$ (p-value = 0.258) nor for outdoor home predictions (p-values: 0.217 and 0.489 for $PM_{2.5}$ of ambient origin and total personal $PM_{2.5}$ respectively).

We also found significant effect modification by age, with subjects younger than 65 years of age having lower calibration coefficients than their older counterparts. Less bias, therefore, would be expected in the health effect estimates from use of surrogate exposures in the health models for older subjects than younger. This effect modification was statistically significant for all combinations, except for that between personal $PM_{2.5}$ of ambient origin and outdoor home model predictions (p-value = 0.06). When the interaction between age and $PM_{2.5}$ concentrations at the nearest ambient monitor was included in Model 1, we were no longer able to detect any between-city heterogeneity for the calibration coefficient for personal $PM_{2.5}$ of ambient origin (p-value = 0.50).The stratified calibration coefficients are presented in Table A3.

Further, we found that estimated calibration coefficients were similar irrespective of the method used to calculate monthly ambient concentrations. Correlations between the two estimation methods were moderate (Spearman r_s = 0.61). When all days within the month were used in the calculation, the calibration coefficient for personal $PM_{2.5}$ of ambient origin was 0.31 (95%CI: 0.14, 0.47), vs. 0.35 (95%CI: 0.26, 0.43) when monthly ambient concentrations were calculated using only those days with personal monitoring. This sensitivity analysis could only be performed for the nearest ambient monitor concentrations, as the outdoor home model predictions were calculated at the monthly level only.

Finally, in a restricted analysis using data only from the five cities for which $PM_{2.5}$ of ambient origin were also available, the calibration coefficients for total personal $PM_{2.5}$ were somewhat higher, although comparable and with overlapping confidence intervals. For the nearest monitor the calibration coefficient was 0.59 (95%CI: 0.35, 0.83), while for the outdoor home model predictions it was 1.05 (95%CI: 0.75, 1.34).

Table S1: By City Summary Statistics of the Exposure Variables. Concentrations are presented in μg/m^3

Cities	Ambient SO_4^{2-}		Personal SO_4^{2-}		Personal $PM_{2.5}$ of ambient origin	
	% missing	Mean (SD)	% missing	Mean (SD)	% missing	Mean (SD)
Atlanta, GA	19.4%	4.8 (1.7)	0.0%	2.6 (1.3)	19.4%	9.7 (4.2)
Baltimore, MD	0.0%	6.7 (4.3)	0.0%	3.3 (2.4)	0.0%	11.0 (4.5)
Boston, MA	0.0%	3.2 (1.1)	0.0%	2.4 (1.2)	0.0%	8.1 (3.2)
Los Angeles, CA						
RTP, NC						
RIOPA						
Los Angeles, CA						
Elizabeth, NJ						
Houston, TX						
Seattle, WA	N/A	N/A	N/A	N/A	88.8%	7.2 (2.5)
Steubenville, OH	0.0%	6.4 (2.5)	0.0%	4.6 (1.8)	0.0%	12.8 (4.2)

Cities	Total personal $PM_{2.5}$		Nearest Monitor $PM_{2.5}$		Model Predicted $PM_{2.5}$	
	% missing	Mean (SD)	% missing	Mean (SD)	% missing	Mean (SD)
Atlanta, GA	0.0%	15.6 (6.5)	0.0%	16.5 (6.6)	0.0%	17.8 (3.2)
Baltimore, MD	0.0%	19.6 (13.8)	8.6%	18.3 (4.8)	8.6%	16.3 (4.2)
Boston, MA	1.8%	15.4 (9.7)	0.0%	14.2 (3.4)	0.0%	13.2 (2.9)
Los Angeles, CA	0.0%	21.0 (13.0)	0.0%	21.1 (7.7)	0.0%	20.4 (4.0)
RTP, NC	0.0%	23.5 (10.8)	0.0%	15.7 (2.8)	0.0%	16.9 (2.9)
RIOPA						
Los Angeles, CA	0.0%	29.3 (14.7)	0.0%	19.8 (6.1)	0.0%	18.9 (5.4)
Elizabeth, NJ	0.0%	45.6 (30.3)	0.0%	15.8 (2.4)	0.0%	15.3 (2.5)
Houston, TX	0.0%	37.3 (23.5)	0.0%	12.8 (3.3)	0.0%	13.4 (2.3)
Seattle, WA	1.1%	12.5 (7.1)	0.0%	11.2 (3.9)	0.0%	10.5 (3.1)
Steubenville, OH	0.0%	18.8 (5.8)	0.0%	20.8 (3.8)	0.0%	19.5 (3.1)

Table S2: Spearman correlation coefficients between exposure variables

Exposures	# observations (# subjects)	Total Personal $PM_{2.5}$	Personal $PM_{2.5}$ of ambient origin	Monitor $PM_{2.5}$	Model Predicted $PM_{2.5}$
Total Personal $PM_{2.5}$	919 (490)	1			
Personal $PM_{2.5}$ of ambient origin	261 (141)	0.66	1		
Monitor $PM_{2.5}$	1029 (502)	0.29	0.51	1	
Model Predicted $PM_{2.5}$	1029 (502)	0.35	0.60	0.86	1

Table S3: Stratified calibration coefficients by season and age

Exposures	$\leq$ 65 yrs		$>$ 65 yrs	
	Summer	Winter	Summer	Winter
Personal $PM_{2.5}$ of ambient origin				
Monitor $PM_{2.5}$	0.10 (-0.03, 0.22)**		0.51 (0.37, 0.65)**	
Model Predicted $PM_{2.5}$†	0.42 (0.27, 0.56)**	0.70 (0.54, 0.85)**	0.42 (0.27, 0.56)**	0.70 (0.54, 0.85)**
Total Personal $PM_{2.5}$				
Monitor $PM_{2.5}$	0.64 (0.24, 1.05)	0.28 (-0.06, 0.62)**	1.19 (0.65, 1.74)	0.82 (0.31, 1.34)
Model Predicted $PM_{2.5}$	0.61 (0.32, 0.91)**		1.33 (0.83, 1.84)	

* p-value$_1$ <0.05, ** p-value$_1$ <0.01 for significant difference from 1

† No significant effect modification by age

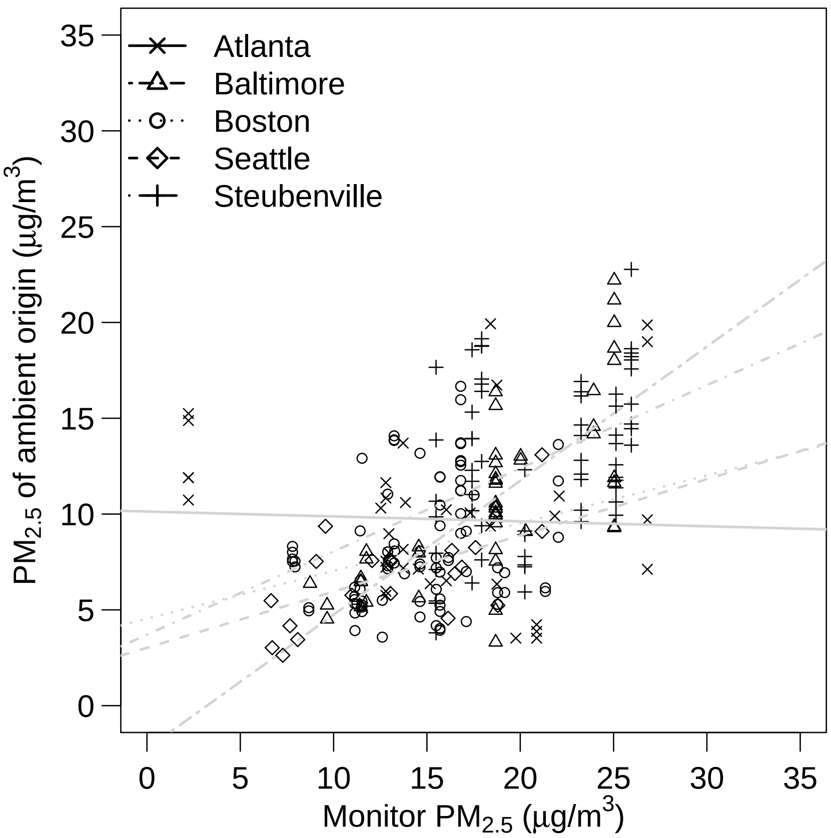

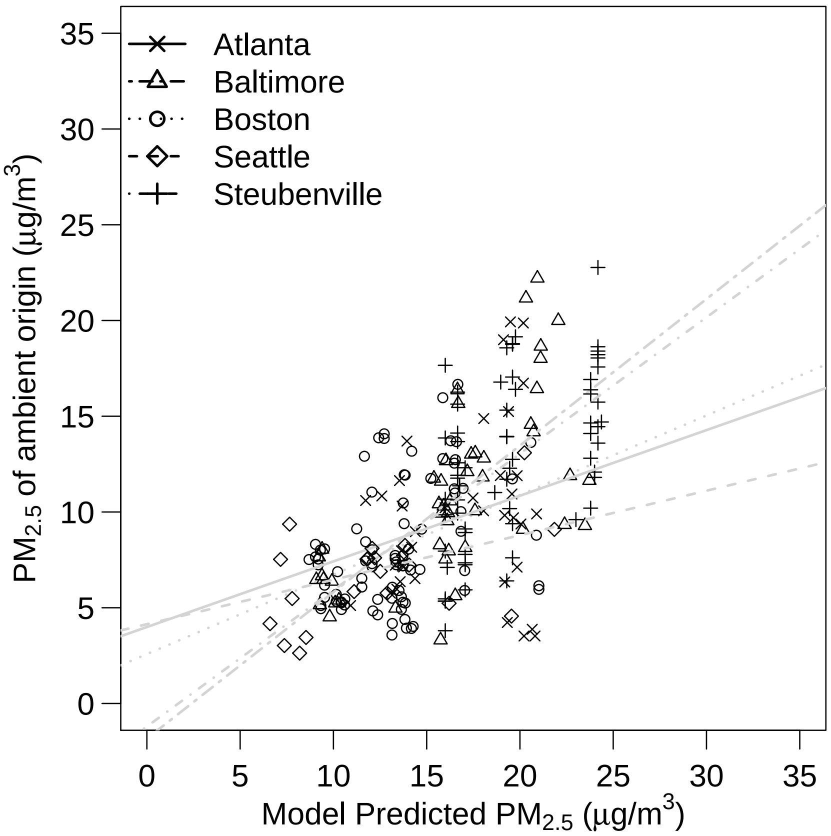

Figure S1: Relationship between monthly personal $PM_{2.5}$ of ambient origin and $PM_{2.5}$ at EPA's monitors (top) and spatio-temporal model $PM_{2.5}$ predictions (bottom).

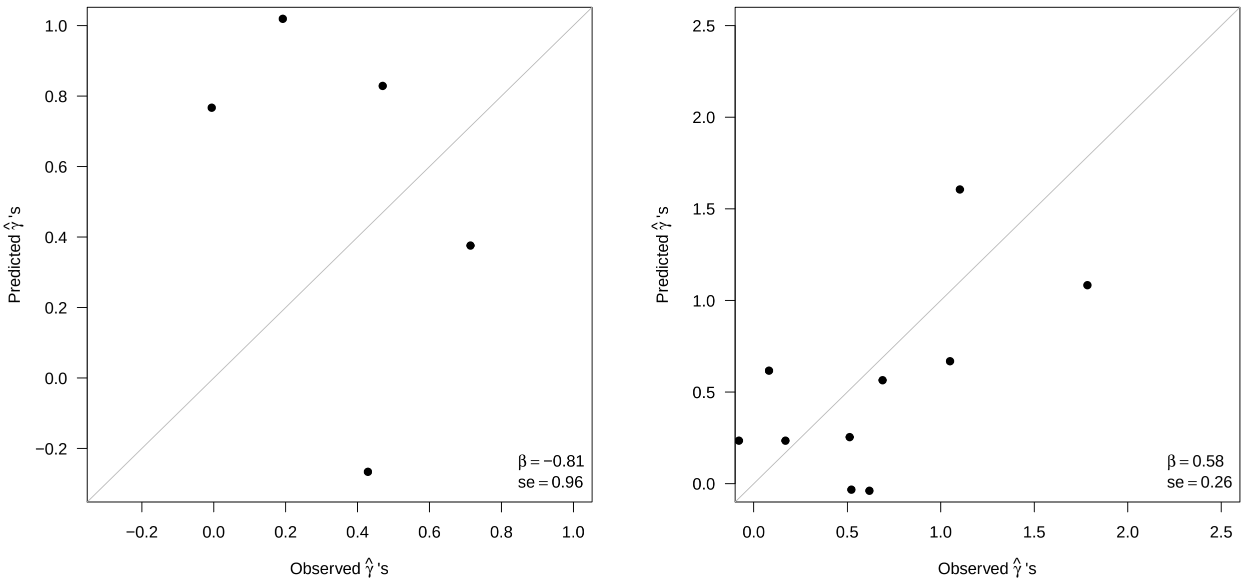

(a) (b)

Figure S2: Cross-validation results for (a) personal $PM_{2.5}$ of ambient origin and (b) total personal $PM_{2.5}$ and nearest monitor concentrations. The gray line represents perfect prediction. The observed city-specific calibration coefficients are on the x-axis, while the predicted city-specific calibration coefficients from the leave one city out procedure are on the y-axis.

Table S4: Summary statistics for the variables considered to explain between-city heterogeneity, averaged across the cities included in our analyses

Variable	Mean	St. Dev.
	Census 2000	
Population	1749622.927	2698534.097
Population density	2506.88	2266.70
Housing unit age	35.18	10.09
# Rooms in housing unit	5.42	0.46
% Of housing units urban	0.91	0.12
# Housing units in structure	7.57	2.92
% Detached homes	0.53	0.13
# People in housing unit	2.56	0.22
% Owners	0.09	0.09
Time people leave home for work	8.41	0.25
% Using public transportation to work	0.07	0.06
% Using car to work	0.85	0.08
% Using kerosene as heating fuel	0.10	0.12
% Using gas as heating fuel	0.54	0.15
% Using electricity as heating fuel	0.31	0.19
# Vehicles per housing unit	1.60	0.18
Minutes to work	29.01	3.29
	Bureau of labor statistics	
Unemployment rate (avg 1998-2002)	4.56	1.20
	American Housing Survey	
% with central AC	0.51	0.33
% with AC in one room	0.12	0.09
	National Climatic Data Center	
Total Annual Cooling Degree Days	1259.80	763.25
Total Annual Heating Degree Days	3858.70	1598.26
Normal Max Temperature (^{o}F)	66.85	6.57
Normal Mean Temperature (^{o}F)	57.66	5.88
Normal Min Temperature (^{o}F)	48.51	5.50
Mean Precipitation (in)	40.80	10.63

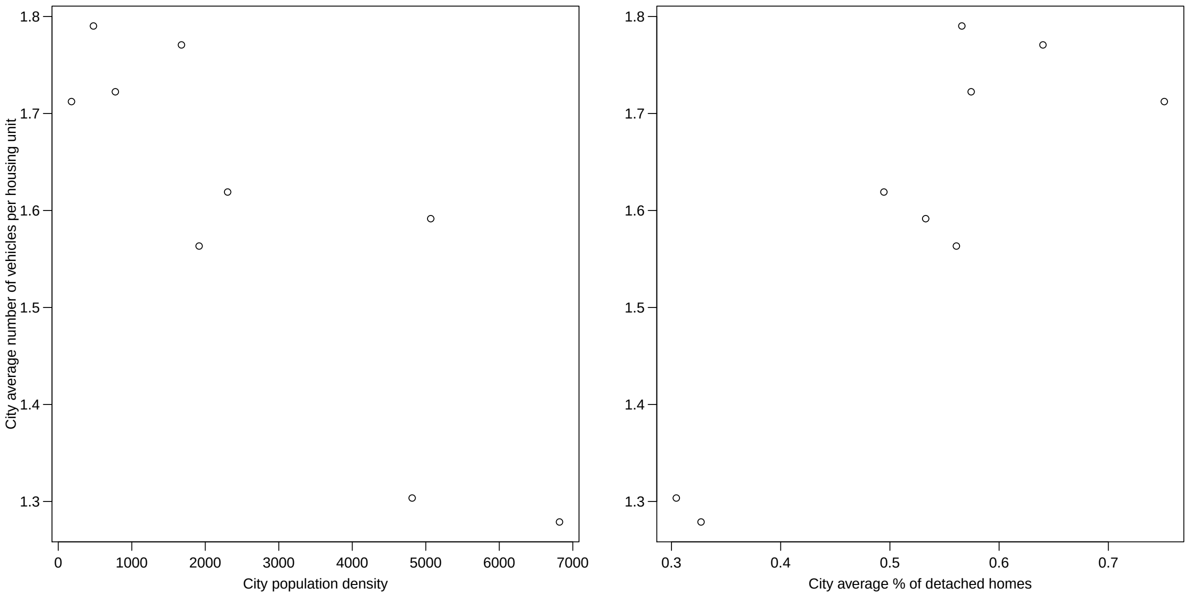

Figure S3: Relationship between the city-average number of cars per housing unit and the population density (left) and the percentage of detached homes in the study area (right).

References

[1] Sarnat SE, Coull BA, Schwartz J, Gold DR, Suh HH: **Factors Affecting the Association between Ambient Concentrations and Personal Exposures to Particles and Gases**. *Environmental Health Perspectives* 2006, **114**(5):649–654.

[2] Koutrakis P, Suh HH, Sarnat JA, Brown KW, Coull BA, Schwartz J: *Characterization of Particulate and Gas Exposures of Sensitive Subpopulations Living in Baltimore and Boston*. Boston, MA: Health Effects Institute 2005. [Research Report 131].

[3] Liu LJ, Box M, Kalman D, Kaufman J, Koenig J, Larson T, et al: **Exposure assessment of particulate matter for susceptible populations in Seattle**. *Environ Health Perspect.* 2003, **111**(7):909–18.

[4] Meng QY, Turpin BJ, Korn L, Weisel CP, Morandi M, Colome S, et al: **Influence of ambient (outdoor) sources on residential indoor and personal $PM_{2.5}$ concentrations: analyses of RIOPA data**. *J Expo Anal Environ Epidemiol* 2005, **15**:17–28.

[5] Sarnat JA, Koutrakis P, Suh HH: **Assessing the Relationship between Personal Particulate and Gaseous Exposures of Senior Citizens Living in Baltimore, MD**. *J. Air & Waste Manage. Assoc.* 2000, **50**:1184 – 1198.

[6] Suh HH, Koutrakis P, Chang LT: *Characterization of the composition of personal, indoor, and outdoor particulate exposures*. Sacramento, CA: California Air Resource Board 2003. [Final Report].

[7] Suh HH, Koutrakis P, Ebelt SE: *Detailed Characterization of Indoor and personal Particulate matter concentrations*. Sacramento, CA: California Air Resource Board 2004. [Final Report].

[8] Suh HH, Zanobetti A: **Exposure error masks the relationship between traffic-related air pollution and heart rate variability**. *JOEM* 2010, **52**(7).

[9] Ward-Brown K, Sarnat JA, Suh HH, Coull BA, Spengler JD, Koutrakis P: **Ambient site, home outdoor and home indoor particulate concentrations as proxies of personal exposures**. *Journal of Environmental Monitoring* 2008, **10**:1041–51.

[10] Ward-Brown K, Sarnat JA, Suh HH, Coull BA, Koutrakis P: **Factors influencing relationships between personal and ambient concentrations of gaseous and particulate pollutants**. *Science of the Total Environment* 2009, **407**:3754–65.

[11] Weisel CP, Zhang J, Turpin BJ, Morandi MT, Colome S, Stock TH, et al: *Relationships of Indoor, Outdoor, and Personal Air (RIOPA): Part I. Collection Methods and Descriptive Analyses*. Boston, MA; Houston, TX: Health Effects Institute; Mickey Leland National Urban Air Toxics Research Center 2005. [Research Report 130].

[12] Williams R, Suggs J, Rea A, Leovic K, Vette A, Croghan C, et al: **The Research Triangle Park particulate matter panel study: PM mass concentration relationships**. *Atmospheric Environment* 2003, **37**:5349–5363.

[13] Williams R, Suggs J, Rea A, Sheldon L, Rhodes C, Thornburg J: **The Research Triangle Park particulate matter panel study: modeling ambient source contribution to personal and residential PM mass concentations**. *Atmospheric Environment* 2003, **37**:5365–5378.
